# Draft Genome Sequences of *Escherichia coli* O104 Strains of Bovine and Human Origin

**DOI:** 10.1128/genomeA.00630-17

**Published:** 2017-08-17

**Authors:** Pragathi B. Shridhar, Isha R. Patel, Jayanthi Gangiredla, Mark K. Mammel, Lance Noll, Xiaorong Shi, Jianfa Bai, Christopher A. Elkins, Nancy Strockbine, T. G. Nagaraja

**Affiliations:** a Department of Diagnostic Medicine and Pathobiology, Kansas State University, Manhattan, Kansas, USA; b Veterinary Diagnostic Laboratory, Kansas State University, Manhattan, Kansas, USA; c Center for Food Safety and Applied Nutrition, Division of Molecular Biology, United States Food and Drug Administration, Laurel, Maryland, USA; d National Center for Emerging and Zoonotic Infectious Diseases, Centers for Disease Control and Prevention, Division of Foodborne, Waterborne and Environmental Diseases, Atlanta, Georgia, USA

## Abstract

Cattle harbor and shed in their feces several *Escherichia coli* O104 serotypes. All O104 strains examined were intimin negative and belonged to the B1 phylogroup, and some were Shiga toxigenic. We report here the genome sequences of bovine O104:H7 (*n* = 5), O104:H23 (*n* = 2), O104:H8 (*n* = 1), and O104:H12 (*n* = 1) isolates and human clinical isolates of O104:H7 (*n* = 5).

## GENOME ANNOUNCEMENT

*Escherichia coli* O104:H4, a hybrid pathotype of enteroaggregative and Shiga toxin-producing *E. coli* (STEC), was responsible for a major foodborne illness outbreak in Germany in 2011 (1). Although cattle are a primary reservoir of STEC, *E. coli* O104:H4 has not been detected in cattle feces. We have reported that cattle harbor and shed O104 serotypes other than those that possess H4 (2). The predominant serotype in cattle feces was O104:H7, and other serotypes included O104:H2, O104:H11, and O104:H21 (2). A majority of O104 serotypes were non-Shiga toxigenic, and only a few strains were Shiga toxigenic, possessing *stx*_1c_. All strains of O104 isolated from cattle feces were *eae* negative, similar to the German outbreak O104:H4 (2). A *stx*_2_-carrying O104:H21 serotype, also negative for *eae*, was involved in an outbreak of hemorrhagic colitis associated with the consumption of raw milk in Helena, MT, in 1994 (3, 4). The O104:H7 serotype has been reported to be associated with sporadic diarrheal cases in humans (5, 6). Whole-genome sequences of the human outbreak strains (O104:H4 German outbreak and O104:H21 Montana outbreak) have been published (7–9). Yan et al. (10) and Lambert et al. (11) have published the whole-genome sequences of *E. coli* O104:H7 strains isolated from cattle feces and of an unknown source, respectively. Here, we report the draft genome sequences of nine bovine *E. coli* O104 strains (five O104:H7, one O104:H8, one O104:H12, and two O104:H23) isolated from cattle feces and five O104:H7 human clinical strains. They are members of the B1 phylogroup, which includes a variety of enteropathogenic, enterohemorrhagic, and Shiga toxigenic strains (12).

Genomic DNA of the target strains was extracted from 1 ml of overnight culture using the Qiagen DNeasy blood and tissue kit (Qiagen, Valencia, CA). The purity of the DNA was assessed spectrophotometrically using a NanoDrop spectrophotometer (Thermo Scientific, Wilmington, DE). Genomic libraries of all the strains were constructed using Nextera XT DNA library preparation kit, and whole-genome sequencing was performed on an Illumina MiSeq sequencer (Illumina, Inc., San Diego, CA) using the MiSeq version 2 reagent kit with 2 × 250 cycles. *De novo* assembly of quality-controlled trimmed sequenced reads was performed using the SPAdes genome assembler version 3.8.2 (http://cab.spbu.ru/software/spades/). A complete list of the strains and their genomic characteristics is provided in Table 1. The genome sequences of *E. coli* O104 strains will further help elucidate the pathogenic potential of *E. coli* O104 serotypes.

**TABLE 1:** Genome characteristics of *E. coli* O104 strains of bovine and human origin

Strain	Serotype	Source	Shiga toxin gene^a^	Genome size (bp)	No. of contigs	GenBank accession no.
2013-6-122E	O104:H7	Cattle feces	*stx*_1c_	5,269,211	329	NEKN00000000
2013-6-148B	O104:H7	Cattle feces	*stx*_1c_	5,302,593	353	NEKL00000000
2013-6-685A	O104:H7	Cattle feces	*stx*_1c_	5,219,160	302	NEKE00000000
2013-6-193B	O104:H7	Cattle feces	—	4,713,172	115	NEKJ00000000
2013-6-289D	O104:H7	Cattle feces	—	5,029,180	122	NEKH00000000
2013-6-380B	O104:H8	Cattle feces	—	4,952,238	83	NEKG00000000
2013-6-210A	O104:H12	Cattle feces	—	4,845,709	152	NEKI00000000
2013-6-173D	O104:H23	Cattle feces	—	5,011,218	71	NEKK00000000
2013-6-140D	O104:H23	Cattle feces	—	5,012,462	91	NEKM00000000
06-3637	O104:H7	Human, clinical	*stx*_2d_	5,044,431	135	NEKS00000000
07-3598	O104:H7	Human, clinical	—	4,885,375	85	NEKQ00000000
08-4061	O104:H7	Human, clinical	*stx*_2a_	5,045,515	120	NEKR00000000
2011C-3665	O104:H7	Human, clinical	*stx*_1c_	5,375,028	340	NEKP00000000
2012C-3400	O104:H7	Human, clinical	*stx*_2a_	4,990,088	151	NEKO00000000

a —, absent.

### Accession number(s).

The whole-genome shotgun sequences have been deposited at DDBJ/ENA/GenBank under the accession numbers listed in Table 1.
